# Synthesis and high sensing properties of a single Pd-doped SnO_2_ nanoribbon

**DOI:** 10.1186/1556-276X-9-503

**Published:** 2014-09-16

**Authors:** Jiang Ma, Yingkai Liu, Heng Zhang, Peng Ai, Nailiang Gong, Ying Zhang

**Affiliations:** 1 Key Laboratory of Yunnan Higher Education Institutes for Optoelectric Information & Technology, Kunming 650500, People's Republic of China; 2 Key Laboratory of Yunnan Normal University for Photoelectric Materials & Device, Kunming 650500, People's Republic of China; 3 Institute of Physics and Electronic Information, Yunnan Normal University, Kunming 650500, People's Republic of China

**Keywords:** Pd-doped SnO_2_, Single nanoribbon, Gas sensor, Ethanol

## Abstract

Monocrystal SnO_2_ and Pd-SnO_2_ nanoribbons have been successfully synthesized by thermal evaporation, and novel ethanol sensors based on a single Pd-SnO_2_ nanoribbon and a single SnO_2_ nanoribbon were fabricated. The sensing properties of SnO_2_ nanoribbon (SnO_2_ NB) and Pd-doped SnO_2_ nanoribbon (Pd-SnO_2_ NB) sensors were investigated. The results indicated that the SnO_2_ NB showed a high sensitivity to ethanol and the Pd-SnO_2_ NB has a much higher sensitivity of 4.3 at 1,000 ppm of ethanol at 230°C, which is the highest sensitivity for a SnO_2_-based NB. Pd-SnO_2_ NB can detect ethanol in a wide range of concentration (1 ~ 1,000 ppm) with a relatively quick response (recovery) time of 8 s (9 s) at a temperature from 100°C to 300°C. In the meantime, the sensing capabilities of the Pd-SnO_2_ NB under 1 ppm of ethanol at 230°C will help to promote the sensitivity of a single nanoribbon sensor. Excellent performances of such a sensor make it a promising candidate for a device design toward ever-shrinking dimensions because a single nanoribbon device is easily integrated in the electronic devices.

## Background

One-dimensional (1D) nanomaterials are attractive building blocks for future high-performance nanoscale devices and sensors [1-3]. With their unique structural characteristics and versatile physical properties, semiconductor nanowires and nanoribbons have been applied to photodetectors [4], nanolasers [5], surface-enhanced Raman scattering (SERS) [6], solar cells [7], sensors, and so on [8,9]. It is well known that 1D nanomaterials possess high surface-to-volume ratio, which is crucial to show high sensitivity [10]. Therefore, special attention has been focused on the application of 1D nanomaterials for detecting toxic, flammable, explosive gases and volatile organic compounds (VOCs). For instance, ZnO-CdS coaxial nanocables have shown to enhance sensitivity toward NH_3_[11]. In_2_O_3_-ZnO core-shell nanowires [12] and Zn doping into the In_2_O_3_ nanowires [13] are found to possess better response toward CO, H_2_, and ethanol. Among these metal oxides and their derivatives, the 1D SnO_2_-based nanomaterial is regarded as a promising candidate for gas monitoring [14].

SnO_2_-based gas sensor has been studied for the detection of a variety of gases. As reported in the literature, the SnO_2_-CeO_2_ nanofiber composite with a Ce content of 6 mol.% exhibited the highest sensor response to ethanol at 250°C [15]. The response to hydrogen of the Pt-decorated bead-like tin oxide nanowire device [16] is approximately 5.7 times higher than that of its undecorated counterpart. Bimetallic Pd/Pt nanoparticle-functionalized SnO_2_ nanowires [17] have a fast response and recovery time to NO_2_. Although 1D SnO_2_-based nanomaterial sensors have been proved to detect many kinds of gases and show high sensitivity to oxidizing and reducing gases, the major drawbacks for detecting VOCs are as follows: (1) They are not selective, i.e., They are not able to distinguish a specified VOC when they are exposed to a mixture of reducing gases [18,19]; (2) the difficulties in using a single nanowire are attributed to the complicated fabrication processes, poor reproducibility, and high costs [20]. In order to remedy these drawbacks or increase the selectivity effectively, various methods are used to improve the sensing properties. One of the best routes to enchance the sensor sensitivity and selectivity is to functionalize the surface of nanowires with rare earth/noble metals, such as Ag [21], Au [22], and Rh [23]. Therefore, it is a good selection to dope 1D SnO_2_ nanomaterials with rare earth/noble metals to optimize their sensing properties.

In this communication, Pd-doped SnO_2_ nanoribbons were synthesized by thermal evaporation. The sensing properties of a single Pd-doped SnO_2_ nanoribbon (Pd-SnO_2_ NB) and its bare counterpart were investigated. It was found that the doping of Pd has a great influence on the electrical properties of the SnO_2_ nanoribbons (SnO_2_ NBs), and that the Pd-SnO_2_ NB sensor possesses reliable, highly sensitive, easily compact, and integrative properties. Our ongoing studies also revealed that doping SnO_2_ with noble or rare earth metals may be a simple and efficient way to improve sensitivity, selectivity as well as response time, and reduce operating temperature. Therefore, it will become an important research field to dope 1D SnO_2_-based nanomaterials with noble or rear earth metals for enchancing their sensing properties.

## Methods

### Synthesis of SnO_2_ nanoribbons and Pd-doped SnO_2_ nanoribbons

The SnO_**2**_ NBs and Pd-SnO_2_ NBs were prepared in a horizontal alundum tube (outer diameter of 4.0 cm, length of 100 cm), which was mounted inside a high-temperature tube furnace. For synthesis of SnO_**2**_ NBs, high-purity SnO_**2**_ powders (>99.99 wt.%) were placed into a ceramic boat, which was then loaded into the central region of the alundum tube. A silicon substrate coated with about 10-nm-thick Au film was put into the tube at a distance of about 10 cm from the ceramic boat. After cleaning the tube several times with nitrogen gas, the tube was evacuated by a mechanical pump to a pressure of 1 to 5 Pa. The temperature at the center of the alundum tube was increased to 1,350°C at a rate of 10°C/min, and it was held at this temperature for 2 h. In the whole experiment, argon was flowed at 30 sccm and the pressure inside the tube was maintained at 125 Torr by continuous pumping. After the furnace was cooled to room temperature, white wool-like products were deposited on the silicon substrates.

For preparation of Pd-SnO_2_ NBs, the starting materials are the mixture of pure SnO_2_ powders (>99.99 wt.%) and Pd(O_2_CCH_3_)_2_ powders mixed in the weight ratio of 20:1, instead of pure SnO_2_ powders. The synthesis procedure is repeated for Pd-SnO_2_ NBs as the above mentioned. After finishing the experiment, Pd-SnO_2_ NBs were obtained.

### Characterization

The nanoribbons were characterized by X-ray diffraction (XRD, D/max-3B Rigaku, Tokyo, Japan) with Cu-K_α_ radiation (*λ* = 0.15406 nm), scanning electron microscopy (SEM) and energy-dispersive spectroscopy (EDS) spectra (SEM Quanta 200 ESEM equipped with EDS from FEI Company, Hillsboro, OR, USA). The microstructures of the obtained samples were analyzed by transmission electron microscopy (TEM) and high-resolution electron microscopy (HRTEM) (JEOL 2010 HRTEM, JEOL, Tokyo, Japan).

### The production of a single nanoribbon device

**Figure 1:**
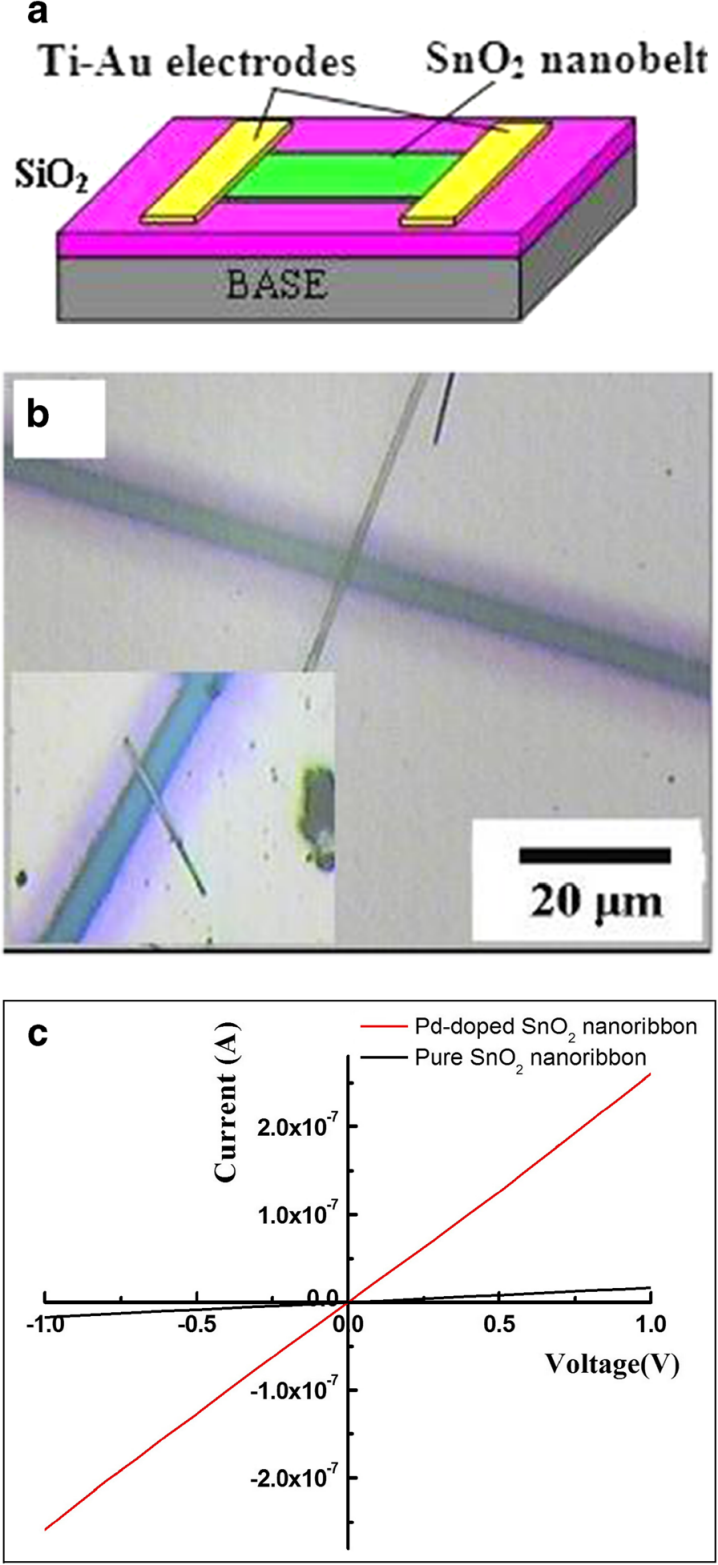
**A single nanoribbon device. (a)** The schematic diagram of the configuration for nanosensors. **(b)** The optical microscopic image of a Pd-SnO_2_ NB; inset: the optical microscopic image of a pure SnO_2_ nanoribbon. **(c)** The *I*-*V* curves of SnO_2_ NB and Pd-SnO_2_ NB.

The single nanoribbon device was represented in the schematic in Figure 1a. For the fabrication of a single-nanoribbon sensor, SnO_2_ NBs/Pd-SnO_2_ NBs were scratched by the tweezers and some products were dispersed in ethanol. Then, a few drops of the resulting suspension were dropped onto a p-type silicon substrate, with a 500-nm-thick SiO_2_ layer, with desired density. Patterned Ti (25 nm) and Au (100 nm) electrodes were successively deposited on the nanoribbons in high vacuum by dual-ion beam sputtering deposition with the assistance of a mesh-grid mask composed of tungsten wires (10 μm in diameter). Since the lengths of the nanoribbons were much larger than the diameter of tungsten wires, the electrodes were formed on the uncovered parts of the nanoribbons. Thus, a Pd-SnO_2_ NB device was obtained and its top-view optical image is shown in Figure 1b. For comparison, a sensor based on a single SnO_2_ NB was also constructed simultaneously, as displayed in the inset of Figure 1b. The calculated results demonstrated that the surface ratio of the Pd-SnO_2_ NB to the pure SnO_2_ NB is 1.04, which is approximately equal to 1 (see Additional file 1 for more details). Figure 1c showed typical *I*-*V* curves when the devices were in air. The approximately linear shape of the curves reveals good ohmic contacts of SnO_2_ NB/Pd-SnO_2_ NB with the electrodes. The colored line slope of the Pd-SnO_2_ NB is larger than that of the SnO_2_ NB device. It suggests that the doped palladium has caused the change of the electrical properties of SnO_2_ NBs and increased the conductance of SnO_2_ NBs 12 times in air at room temperature.

### Gas test

The measurement was processed with a static method. The sensor on a heating station with a precision temperature controller was put into a closed stainless steel chamber and the predetermined amount of solvent was injected into the chamber for the measurement of the sensing performance. The chamber was also equipped with a fan and an evaporator. The evaporator was used to accelerate the volatilization of the VOC liquid, and the fan was employed to obtain a homogeneous gas mixture in the chamber [24]. The sensing properties were measured by the Keithley 4200 semiconductor testing system (Keithley Instruments, Inc., Cleveland, OH, USA). The testing bias voltage was 1 V and the testing interval was 3 min.

## Results and discussion

### Crystal structure and morphology

**Figure 2:**
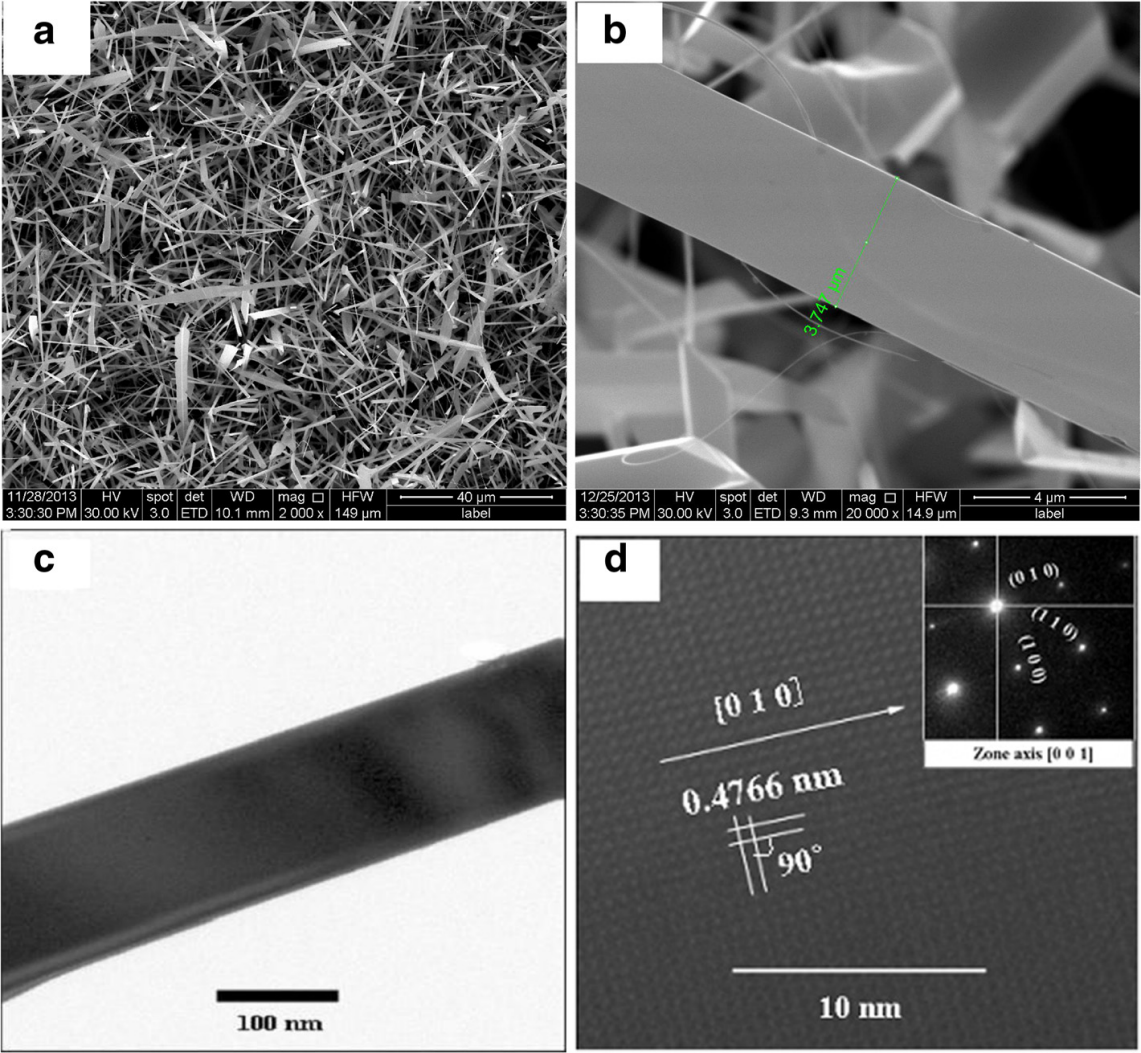
**SEM and HRTEM images of tin oxide nanoribbons doped with palladium. (a)** SEM image of Pd-SnO_2_ NB at low magnification. **(b)** SEM image of Pd-SnO_2_ NB at high magnification. **(c)** TEM image of a Pd-SnO_2_ NB. **(d)** HRTEM image of a Pd-SnO_2_ NB and its SAED shown in inset.

Figure 2a shows the morphology of the synthesized materials. It is evident that there are lots of nanoribbons with a thickness of 50 to 60 nm, widths of 2 to 4 μm, and lengths of up to 50 μm. Their widths are uniform along the length. Figure 2b displays a single Pd-SnO_2_ NB at high magnification with a smooth surface and a width of 3.747 μm.

Further insight into the structure of NBs is obtained from TEM and HRTEM recorded on an individual Pd-SnO_2_ NB. The low-magnification TEM image of a typical Pd-doped SnO_2_ NB is presented in Figure 2c. It shows that the nanoribbon is straight with a uniform thickness. The HRTEM in Figure 2d exhibits good crystalline and continuous lattice fringes over a large area, which is acquired by enlarging Figure 2c. The interplanar spacing is 4.766 Å, corresponding to the d (100) interplanar spacing for the tetragonal structure SnO_2_. Its selected-area electron diffraction (SAED) pattern is shown in the inset of Figure 2d, which shows the lattice plane index and zone axis [001].

**Figure 3:**
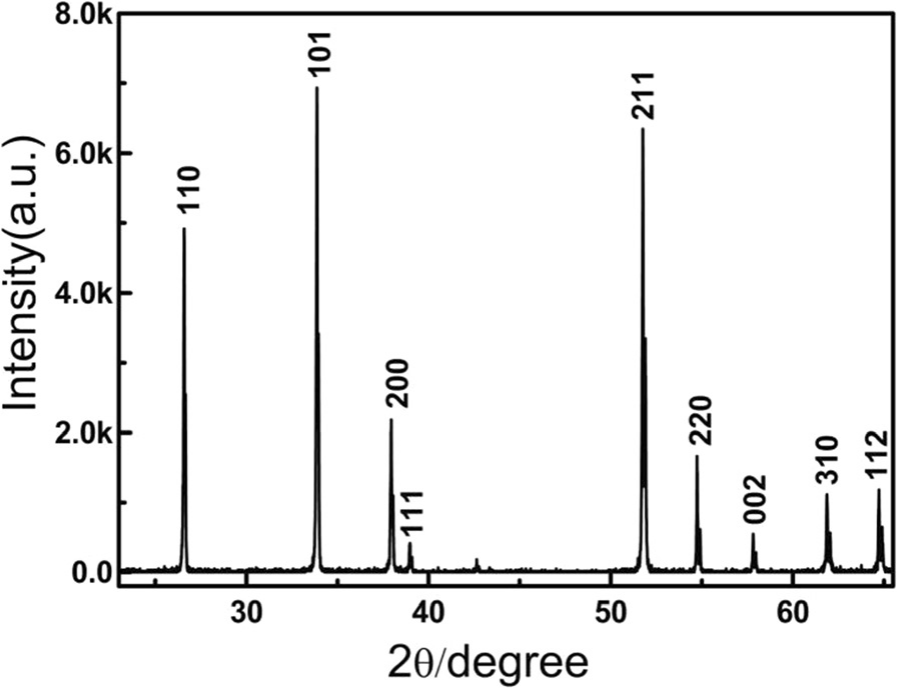
**XRD patterns of Pd-SnO**_**2 **_**NBs.**

**Figure 4:**
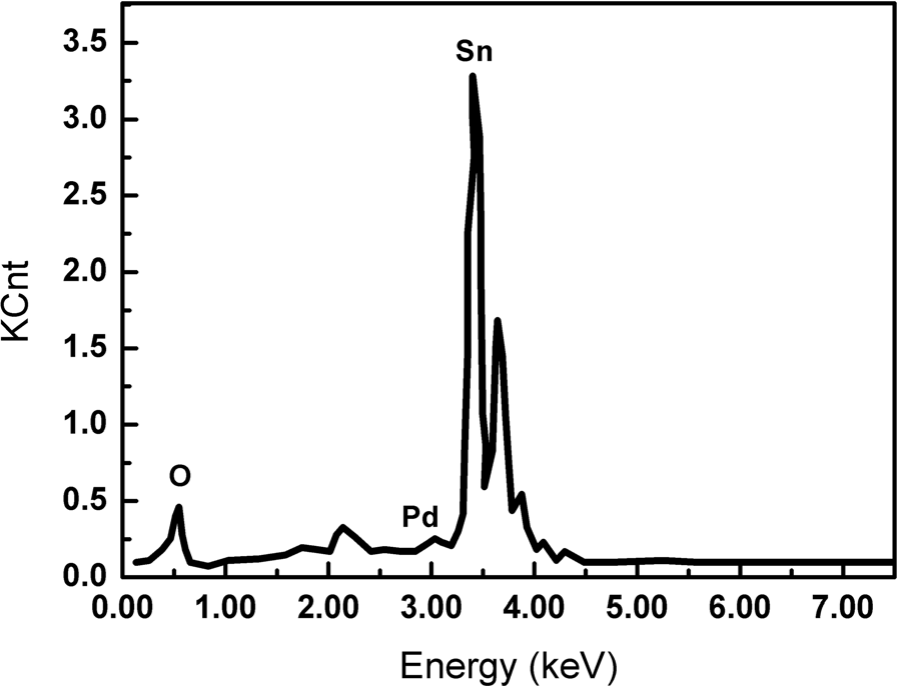
**EDX image of a single Pd-SnO**_**2 **_**NB.**

The X-ray diffraction pattern of the obtained Pd-SnO_2_ NBs is presented in Figure 3. All diffraction peaks can be perfectly indexed as the tetragonal rutile SnO_2_ structure (JCPDS card No. 02-1340). The lattice constants of Pd-SnO_2_ NBs calculated from the XRD data are *a* = *b* = 0.4736 nm and *c* = 0.3188 nm. No diffraction peaks of other materials can be observed, indicating that the doping of Pd element does not cause the change of crystal structures. To detect whether Pd is doped into the SnO_2_ NBs or not, EDS analysis of a single Pd-SnO_2_ NB is implemented, as shown in Figure 4. The EDS demonstrates that the synthesized Pd-SnO_2_ NB are composed of Sn, O, and Pd. Analysis gives that the amount of doped Pd is 0.8 wt.%.

### Analysis of gas sensing

The sensor response (*R*_s_) is defined as follows:

$$R_{s}=R_{a}/R_{g}$$

where *R*_a_ is the sensor resistance in air (base resistance) and *R*_g_ is the resistance in a mixture of target gas and air. The response time and recovery time are defined as the time taken by the sensor to achieve 90% of the total resistance change in the case of adsorption and desorption, respectively [15].

**Figure 5:**
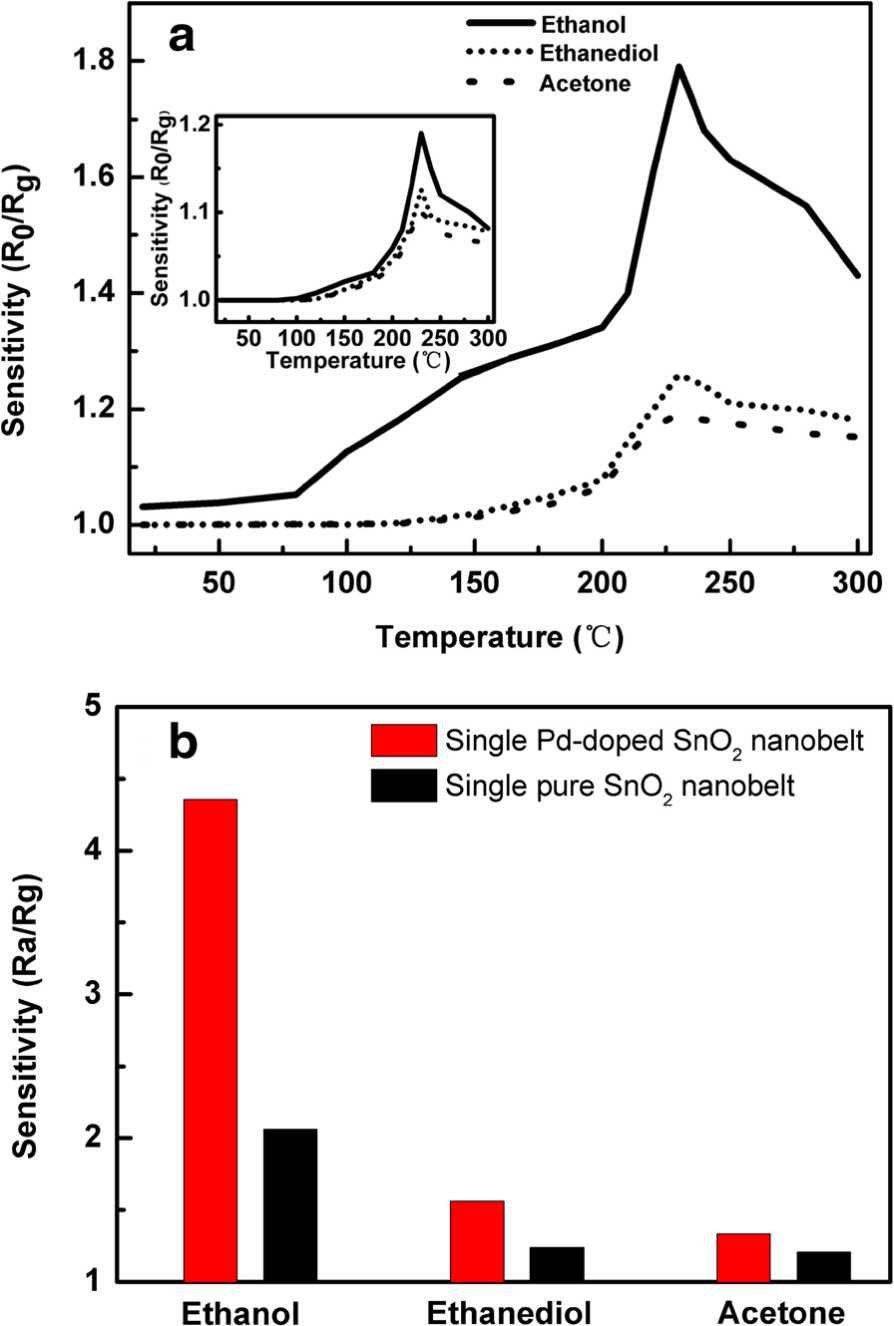
**Sensitive comparison of a single SnO**_**2 **_**nanoribbon with Pd-doped SnO**_**2 **_**nanoribbon for ethanol, ethanediol, and acetone gases. (a)***R*_s_ versus T curves of a Pd-SnO_2_ NB to ethanol at 100 ppm; *R*_s_ versus T curves of a SnO_2_ NB to ethanol at 100 ppm in inset. **(b)** The bar diagram of two devices responding to different gases at 230°C.

In order to determine the optimum working temperature, the responses of the fabricated devices to ethanol, ethanediol, and acetone vapors at 100 ppm were tested as a function of the operating temperature in the range of 25°C ~ 300°C, as shown in Figure 5a and its inset for a single Pd-SnO_2_ NB and its undoped counterpart, respectively. It is obvious that the optimum operating temperature of the two devices to three tested volatile organic compounds, are at 230°C. Compared with the higher optimum working temperatures in previous reports [15-17], the reduction of optimal operating temperature and the enhanced response within the temperature range of 25°C ~ 230°C can be ascribed to the catalytic activation upon Pd doping into SnO_2_, which promotes the catalytic process itself that diminishes the activation energy needed for a chemical reaction to correspond at low temperatures [25].

Selectivity is a key parameter of gas sensors. Figure 5b shows the response bar diagram of the two devices to various target gases (1,000 ppm), including ethanol, ethanediol, and acetone at 230°C. It should be noted that the Pd-SnO_2_ NB exhibits an outstanding selectivity to ethanol at 230°C and its sensitivity is 4.3, which is 2.2 times as much as that of the SnO_2_ NB. Hence, the highest sensing response of the Pd-SnO_2_ NB sensor is to ethanol.

**Figure 6:**
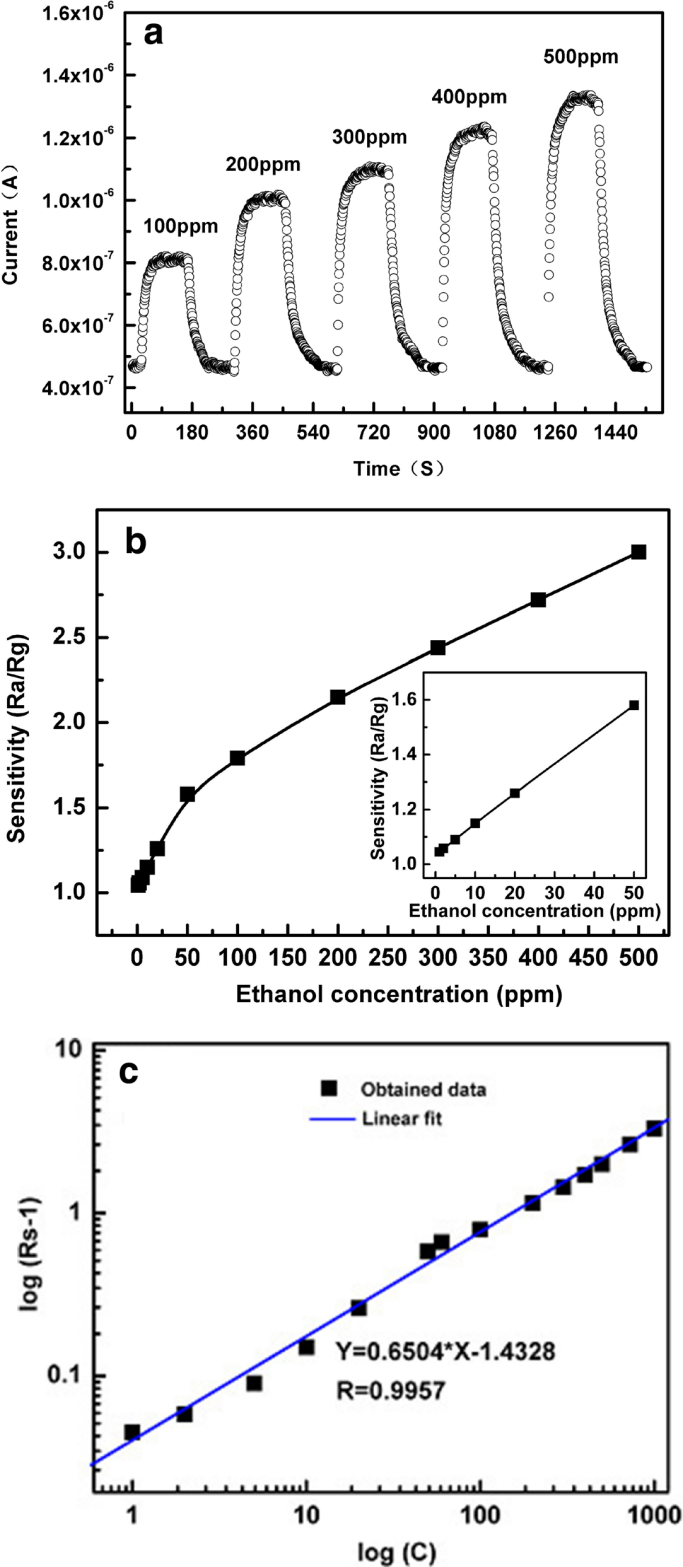
**Sensitive responses to ethanol of a single Pd-doped SnO**_**2 **_**nanoribbon tested at 230°C. (a)** Responses of a Pd-SnO_2_ NB to ethanol at 100 ~ 500 ppm at 230°C. **(b)** Responses of a Pd-SnO_2_ NB to ethanol at 1 ~ 1,250 ppm at 230°C. **(c)** The log(*R*_s_ - 1) versus log(*C*) curve and its fitted line.

To investigate the sensing property of ethanol vapor-exposed Pd-SnO_2_ NB sensor under different analyte concentrations at 230°C, the ethanol concentration was tuned from 100 ppm to 500 ppm, and the sensing characteristics were recorded in Figure 6a. It was found that the response increased from 1.8 to 3 with an increase in ethanol concentration. The typical behavior of the Pd-SnO_2_ NB sensor to different concentrations of ethanol is identical to that of the empirical model [26]. In contrast, for the pure SnO_2_ NB, the current increased from 1.4 × 10^-7^ to 2.4 × 10^-7^ A (figure is not shown here), and its response was 1.7 when the concentration of ethanol reached 500 ppm. It confirms that the Pd-SnO_2_ NB sensor has much improvement compared to its counterpart.

We provide more details of the response to 1 ~ 1,250 ppm of ethanol concentrations in Figure 6b. It is observed that the sensor response depended on the approximate linearity on ethanol vapor concentration in the ranges of 1 to 60 and 60 to 1,000 ppm. More importantly, the slope of the curve with concentrations varying from 1 to 60 ppm in the inset of Figure 6b is larger than that obtained from 60 to 1,000 ppm. This result indicates that the Pd-SnO_2_ NB sensor is suitable for quantitative detection for ethanol at low concentrations, greatly simplifying the use in practical terms. On the other hand, it was observed that the knee responds as the concentration of ethanol equals to 60 ppm. Many research literatures observed this phenomenon in the concentration range of 10 to 50 ppm [25,27], but few people discussed this phenomenon. To the best of our knowledge, the turning point of alcohol concentration when it reaches 60 ppm on the Pd-SnO_2_ NB sensor is reported for the first time. The appearance of the inflection point may help us to establish theoretical systems and boost the effect in practical application. At the same time, the response of gas sensors based on metal oxide semiconductors is empirically represented as *R*_s_ = *a*[*C*]^*b*^ + 1 [27] at a certain working temperature, and then the above equation can be rewritten as log(*R*_s_ - 1) = *b*log(*C*) + log*a*, where *a* and *b* are constants and *C* is the concentration of the target gas. In this current work, log(*R*_s_ - 1) exhibits a linear relationship with log(*C*): *Y* = 0.6504*X* - 1.4328, enjoying a good relativity (*R* = 0.9957), as shown in Figure 6c. Therefore, practical ethanol sensors using these SnO_2_ NBs can be greatly simplified in detection, due to the linear relationship between S and C obtained in logarithmic forms.

**Figure 7:**
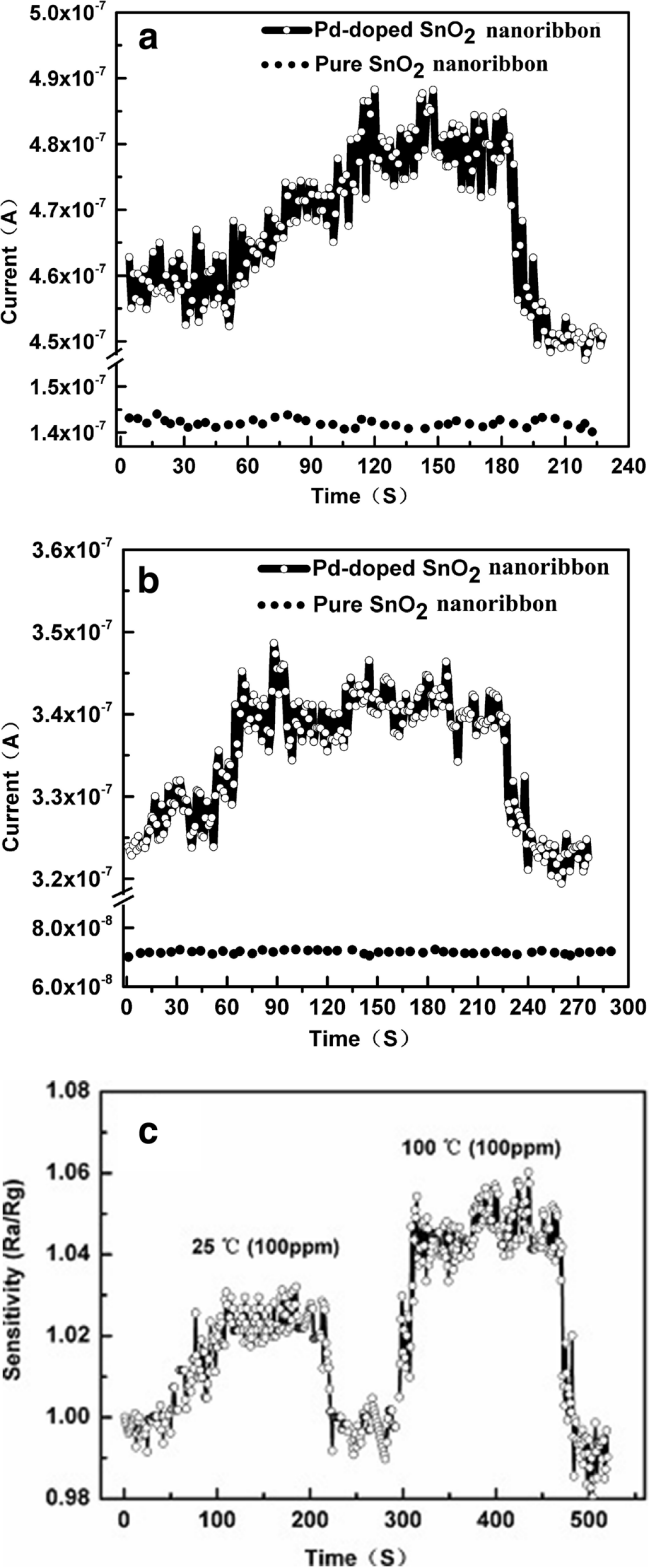
**Sensitive responses to different ethanol concentrations of single Pd-doped SnO**_**2 **_**nanoribbon and SnO**_**2 **_**nanoribbon at different temperatures. (a)** Responses of two devices to 1 ppm of ethanol at 230°C. **(b)** Responses of two devices to 100 ppm of ethanol at 100°C. **(c)** Responses of a Pd-SnO_2_ NB to 100 ppm of ethanol at 25°C and 100°C.

We further explore the response of the device under low concentration of ethanol gas at low temperature. Original SnO_2_ NBs almost have no response to 1 ppm ethanol, but Pd-SnO_2_ NBs have the response of 1.045 and exhibit good stability at 230°C, as shown in Figure 7a, which is more sensitive than that of the minimum concentration of 2 ~ 10 ppm reported [28].

For low temperature detection, the response of a single Pd-SnO_2_ NB is 1.056 as the concentration of ethanol vapor is 100 ppm at 100°C whereas pure SnO_2_ NB has no response, as shown in Figure 7b,c. It follows from our experimental results that the single Pd-SnO_2_ NB may work steadily at 100°C for 100 ppm ethanol at low temperature. Interestingly, the single Pd-SnO_2_ NB has a relatively stable response to ethanol of 100 ppm at 25°C and its sensitivity is nearly 1.02. For a low temperature, even around room temperature, effective gas detection such as ethanol gas has been a direction that the scientists are trying to conquer. However, a single Pd-SnO_2_ NB may achieve this goal.

The response (recovery) time can provide the dynamic response of the sensors upon adsorption and desorption, respectively, which is an important parameter for electronic sensors. The response (recovery) time of the Pd-SnO_2_ NB device and its counterpart were measured and listed in Table 1. At first glance, the two sensors show good sensitivity to three kinds of organic compounds and have relatively faster response (recovery) time, which could be attributed to the monocrystalline SnO_2_ films compared to polycrystalline ones [23,27,28] based on the fact that our prepared NBs are single crystals obtained from the XRD and HRTEM results. It is also found that the response time is shorter with increasing temperature. The Pd-SnO_2_ NB has a quick response (recovery) time of 8 s (9 s) to ethanol at 300°C. This phenomenon has been explained as a speeding up of kinetics of a gas-surface reaction at a higher temperature and a shorter time for C_2_H_5_OH absorbing or desorbing from the nanoribbons [25].

**Table 1:** The performance of two kinds of NB sensors

**Test gas**	**Temperature**	**A single Pd-SnO**_**2 **_**NB**			**A single pure SnO**_**2 **_**NB**		
**(100 ppm)**	**(°C)**	**Sensitivity (*R***_**a**_**/*R***_**g**_**)**	**Response time (s)**	**Recovery time (s)**	**Sensitivity (*R***_**a**_**/*R***_**g**_**)**	**Response time (s)**	**Recovery time (s)**
Ethanol	230	1.80	18	28	1.19	19	26
	300	1.43	8	9	1.082	8	15
Ethanediol	230	1.31	18	20	1.12	20	26
	300	1.22	9	10	1.078	9	16
Acetone	230	1.17	24	30	1.102	12	15
	300	1.16	14	11	1.065	9	14

During the whole measurement process, similar measurements had been carried out every day in the first seven days, and the performance of the sensor is stable and distinguished. After that, we measured once every 10 days and the performances of the sensors were also stable and reliable. Up to date, it has been several months.

### Sensing mechanism

As an n-type semiconductive oxide, its sensing response depends on grain size, porosity, lattice defects, activation energy of adsorption of the test gases on its surface, the quantity of oxygen adsorption, and so on. For the purpose of the sensing response mechanism of the Pd-SnO_2_ NB sensor, we will return to its microstructure and electron transfer process. It is well known that the surface of the nanoribbon sensor is covered with chemisorbed oxygen ions such as O^-^, O_2_^-^, and O_2_^2-^. When temperature is elevated, the reactions can be written as follows [29]:

(1) $$O_{2}\left(\text{ads}\right)+e^{-}↔{O_{2}}^{-}\left(\text{ads}\right)$$

(2) $${O_{2}}^{-}\left(\text{ads}.\right)+e^{-}↔2O^{-}\left(\text{ads}\right)$$

(3) $$O^{-}\left(\text{ads}\right)+e^{-}↔O^{2-}\left(\text{ads}\right)\text{.}$$

For Equation 1, it mainly happens within the temperature range of 25°C to 150°C, and the reaction of Equation 2 mainly happens from 150°C to 300°C [30]. The interactions between ethanol and lattice oxygen of the Pd-SnO_2_ NB can be described as follows [31]:

(4) $$3{O_{2}}^{-}+C_{2}H_{5}OH_{\left(g\right)}=3H_{2}O_{\left(g\right)}+\;2CO_{2\left(g\right)}+\;3e^{-}$$

(5) $$6O^{-}+\;C_{2}H_{5}OH_{\left(g\right)}=\;3H_{2}O_{\left(g\right)}+2CO_{2\left(g\right)}+6e^{-}$$

(6) $$6O^{2-}+\;C_{2}H_{5}OH_{\left(g\right)}=3H_{2}O_{\left(g\right)}+2CO_{2\left(g\right)}+12e^{-}$$

**Figure 8:**
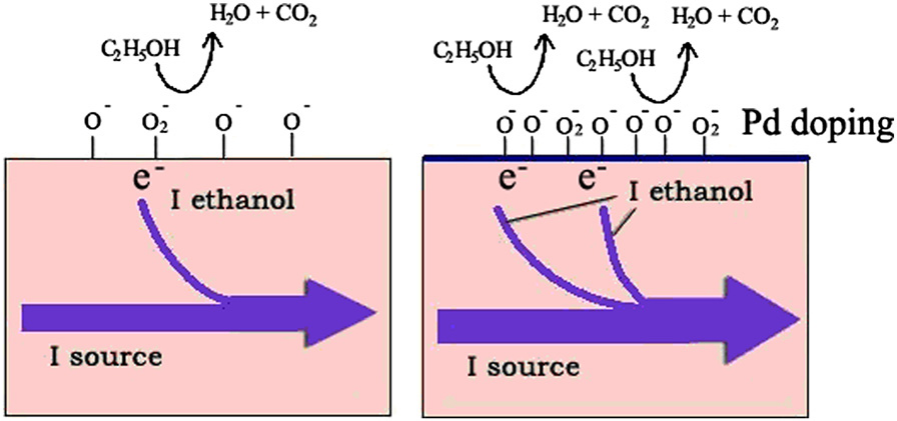
**Illustrations of the sensing mechanism on SnO**_**2 **_**NB and Pd-SnO**_**2 **_**NB.**

For SnO_2_ and Pd-SnO_2_ NBs, their eventual current is composed of source current and sensitive response current, as depicted in Figure 8. The current of a Pd-SnO_2_ NB is heavier than that of a pure SnO_2_ NB. This reveals that 0.8 wt.% of palladium doping may, on one hand, catalytically enhance the gas-sensing current [31]. On the other hand, oxygen vacancies and/or electron donor states would be expected to be produced with Pd doping, which can strengthen oxygen adsorption on the surface of SnO_2_ NB [32] so that the doped nanoribbon will show higher and better sensitivities.

## Conclusions

In summary, pure SnO_2_ NBs and Pd-SnO_2_ NBs have been successfully synthesized by thermal evaporation at 1,350°C, and a highly sensitive single Pd-SnO_2_ NB and SnO_2_ NB sensor devices have been developed. The sensing properties of the two devices were investigated systematically. It is found that the single pure SnO_2_ NB sensor shows a high sensitivity at 230°C to ethanol, and the Pd-SnO_2_ NB one exhibits a higher sensitivity of 4.3 to ethanol at the concentration of 1,000 ppm, which is the highest sensitivity for a single SnO_2_ NB. The Pd-SnO_2_ NB can detect C_2_H_5_OH from 25°C to 300°C for a wide range of concentration (1 ~ 1,000 ppm). Additionally, whether it is ethanol, ethanediol, or acetone, the Pd-SnO_2_ NB shows a better gas-sensing property than the pure SnO_2_ NB. Furthermore, both at low temperature and low concentration, the response of Pd-SnO_2_ NB is better than that of its counterpart.

The ethanol nanosensors described have low power consumption which can respond to as low as 1 ppm of ethanol at 100°C and are easily integrated because of its nanoscale size. In addition, a single nanoribbon/nanobelt or nanorod device with a single crystal structure has no composition segregation and other advantages such as reliable and sensitive properties, low weight, and relatively quick response and recovery time. Hence, it offers a unique route toward miniaturization of sensors while maintaining their functionality and opens up a new horizon for sensor design. Therefore, this kind of gas sensor can be applied to a variety of applications, including leak detection of hydrocarbon fuels, personal health monitoring and environmental monitoring.

## Competing interests

The authors declare that they have no competing interests.

## Authors' contributions

YL guided the experiments and test process and revised the paper. JM carried out the synthesis of nanoribbons, gas sensitive test, and prepared the manuscript. PA and NG carried out the characterization. HZ and YZ analyzed the data. All authors read and approved the final manuscript.

## Supplementary Material

Additional file 1 Supporting information. Click here for file
