# Supplementary material for: Synthesis and high sensing properties of a single Pd-doped SnO2 nanoribbon
Source: Nanoscale Res Lett. 2014 Sep 16;9(1):503. doi: 10.1186/1556-276X-9-503 (PMC4181503; doi:10.1186/1556-276X-9-503)

**Supporting Information**

The single Pd-SnO_2_ nanoribbon device and its counterpart (pure SnO_2_ nanoribbon device) were shown in Fig.S1(a) and Fig. S1 (b). Their high magnification images were separately shown in Fig. S1 (c) and Fig. S1 (d). The two nanoribbons possess the same thickness of 60 nm. The the surface ratio of the Pd-SnO_2_ NB to the pure SnO_2_ NB is calculated as follows: for Pd-SnO_2_ device, S _Pd surface area_ = 5.6μm (length) × 1.8μm (width)+ 0.06μm × 5.6μm × 2= 10.75μm^2^ ; for the counterpart, S_surface area_= 6.8μm (length) × 1.4μm (width)+ 0.06μm × 6.8μm × 2= 10.34μm^2^. Therefore, The ratio of their surface areas is 1.04.


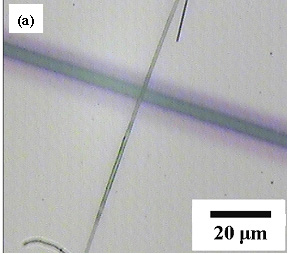


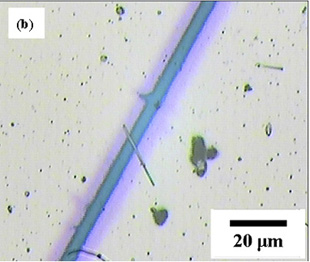


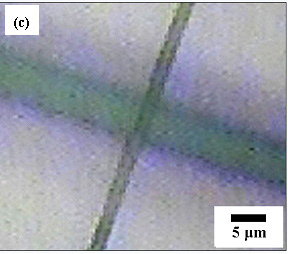


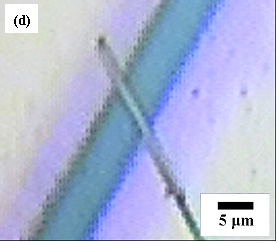

Supplement: Additional file 1 — Supporting information. [file 1556-276X-9-503-S1.doc]
